# Relationship between optimism, emotional intelligence, and academic resilience of nursing students: the mediating effect of self-directed learning competency

**DOI:** 10.3389/fpubh.2023.1182689

**Published:** 2023-05-18

**Authors:** Eun Hee Hwang, Kon Hee Kim

**Affiliations:** ^1^Department of Nursing, Wonkwang University, Iksan, Republic of Korea; ^2^College of Nursing, Ewha Womans University, Seoul, Republic of Korea

**Keywords:** academic resilience, emotional intelligence, optimism, self-directed learning competency, nursing student

## Abstract

**Background:**

The evolution toward future education following the 4th industrial revolution and the coronavirus disease 2019 (COVID-19) pandemic have changed nursing education dramatically. Online classes have become a new paradigm of education, and are expected to develop and be maintained in various forms even after the end of COVID-19. Therefore, attention is focused on finding ways to improve learners’ achievements in a distance learning environment. This study aimed to examine the mediating effects of self-directed learning competency on the relationships between optimism, emotional intelligence and academic resilience among nursing students.

**Methods:**

A cross-sectional descriptive design was conducted using convenience sampling of 195 nursing students in South Korea. Data were analyzed with descriptive statistics, Pearson’s correlation coefficients, multiple regression, and mediation analysis using SPSS/WIN 26.0 program.

**Results:**

There were significant positive correlations among self-directed learning competency, optimism, emotional intelligence and academic resilience. The self-directed learning competency acts as a mediator in explaining relationship between optimism, emotional intelligence and academic resilience, respectively.

**Conclusion:**

This study provides the evidence for the role of self-directed learning competency in the relationship between optimism, emotional intelligence and academic resilience in nursing students. Rapid changes in education are inevitable due to changes in clinical settings and the impact of repeated infectious disease outbreaks including the COVID-19 pandemic. This study suggests strengthening positive psychology and self-directed learning capability of nursing students as a strategy to prepare for changes in education and clinical areas.

## 1. Introduction

With changes in the healthcare environment and demand, expectations for nursing competency have increased in the clinical field and have led to the demand for a change in the quality of nursing education. In particular, the coronavirus disease 2019 (COVID-19) pandemic has led to a paradigm shift in the education system, and it has become necessary to redefine measures to improve nursing students’ academic achievement. During the transition to non-face-to-face education, attributable to the pandemic, medical students experienced high levels of stress, depression, and anxiety due to academic burnout (1, 2). As the post-COVID-19 era begins, it is time to explore strategic changes in higher education in nursing.

Academic resilience is an affective factor defined as students’ ability to overcome academic stress or pressure (3). Academic resilience is an individual’s ability to achieve academic success even during difficult circumstances (4, 5). For nursing students, academic resilience is a protective factor for coping with stress due to clinical practice and unexpected crises in unfamiliar environments (6, 7). Therefore, the future direction of nursing education must focus on promoting and strengthening academic resilience.

Positive psychology is a new perspective within psychology. In particular, there is increasing interest in optimism about personal characteristics that help undergraduates adjust to school life and develop individually. Optimism is a tendency to expect positive outcomes in the future and assign positive meaning to reality. It can be seen as the confidence or self-belief that helps a person handle everyday situations efficiently, and this disposition reduces negative stressors and is enhanced through learning during development (8). Optimistic emotions enable a student to overcome difficulties during the learning process with positive thinking and to become more engaged in the learning process (9); it is the most influential variable that explains nursing students’ satisfaction with their major (6). Emotional intelligence, the ability to understand others’ emotions and regulate and control one’s own emotions (10), is also a factor that influences academic achievements and motivation (11) and can be developed through education and training. Therefore, it is necessary to train nursing students to combine optimism and emotional intelligence to control their academic achievements, implying the need for exploring changes in educational methods by determining and developing the strengths of learners.

Self-directed learning refers to the initiative to judge learning needs, establish learning goals, select and implement appropriate learning strategies, and evaluate learning outcomes with or without help from others (12). In nursing education, self-directed learning is an effective way for students to apply their vocational skills in various clinical settings after graduation and also cope with the rapidly changing healthcare environment and demonstrate their professional competencies (13, 14). However, Korean nursing students have moderate self-directed learning skills (15). They do not have many opportunities to learn autonomously because as high school students, they prepare for university entrance exams following the conventional academic process and schedule; and as nursing students, they must complete a curriculum that combines theory and practical education to succeed in a national exam. During the COVID-19 pandemic, learning activities centered on learner-driven participation, and this task is also applicable to nursing education. Therefore, education that fosters learners’ self-directed learning competency should be introduced in nursing education to eventually improve the level of academic achievement.

This study proposes a conceptual framework of the relationship between variables, based on prior studies, to identify the mediating effects of self-directed learning competency on the relationship between optimism, emotional intelligence, and academic resilience in nursing students. Prior research confirms the correlation between optimism and academic resilience in nursing students (6) and emotional intelligence and academic resilience in undergraduates (16). Resilience enables nursing students to overcome stressful situations during their course curriculum and practical training and can help them achieve academic success and employment (17). Correlations have also been found between nursing students’ negative emotions and self-directed learning competency (2) and emotional intelligence and self-directed learning competency (18). Self-directed learning competency affects optimism and emotional intelligence, both of which impact academic resilience; hence, this study conceptualized that self-directed learning competency mediates the relationship between optimism, emotional intelligence, and academic resilience.

In particular, the academic resilience of nursing students, which had been maintained at a moderate level before the COVID-19 pandemic (6, 7) in 2020, when the education form was completely switched to non-face-to-face classes due to the COVID-19 pandemic was rapidly degraded (19). Therefore, it is necessary to check how optimism, emotional intelligence, and self-directed learning competency, which were identified as significant influencing factors on the academic resilience of nursing students, changed under the COVID-19 pandemic situation.

Accordingly, this study examines the relationship between optimism, emotional intelligence, self-directed learning competency, and academic resilience to validate the mediating effects of self-directed learning competency for developing programs that promote academic resilience in nursing students. The specific study objectives are to identify participants’ general characteristics and levels of optimism, emotional intelligence, self-directed learning competency, and academic resilience; to identify differences in academic resilience based on participants’ general characteristics; to identify correlations between optimism, emotional intelligence, self-directed learning competency, and academic resilience; to verify the mediating effect of self-directed learning competency in the relationship between optimism and academic resilience; and, to verify the mediating effect of self-directed learning competency in the relationship between emotional intelligence and academic resilience.

## 2. Participants and methods

### 2.1. Design

This descriptive correlation study examined the effect of optimism and emotional intelligence on academic resilience through the mediating effect of self-directed learning competency in nursing students.

### 2.2. Participants

The authors collected data through convenience sampling with a population of undergraduate nursing students who had taken online classes for the past 2 years during the COVID-19 pandemic. After posting study-related recruitment documents on the SNS for nursing students, students who voluntarily decided to participate in the study were provided with a link to the Google survey along with an explanation describing the purpose of the study, procedures, and the rights of participants. Nursing students who decided to participate in the study were allowed to respond by clicking on the link. The minimum sample size required for regression analysis with a significance level of 0.05, medium effect size, power of 0.95, and 10 predictors was determined as 172 using the G*power program 3.1.9.2. A total of 205 students were recruited, considering the dropout rate of 20%, and data collected from 195 students were analyzed, excluding 10 cases with insufficient responses.

### 2.3. Instruments

The Korean version instruments consisted of general characteristics including age, gender, academic year, economic status, school life stress, subjective health status, major satisfaction, motivation for applying to nursing, etc., the Life Orientation Test-Revised, the Wong and Law Emotional Intelligence Scale, and the Self-directed Learning Competency, and the Academic Resilience Scale.

#### 2.3.1. Optimism

The Life Orientation Test-Revised (LOT-R), which is Carver and Bridges’ revision (20) of the Life Orientation Test (LOT) developed by Scheier and Carver (21) and is translated and verified by Shin (22) was used for measuring optimism. This scale comprises 10 items regarding general expectations for a positive life. It has three positive items, three negative items, and four filler items that prevent respondents from recognizing that the questionnaire intends to measure optimism. Each item is scored on a 5-point Likert scale. The negative items are reverse scored, and the filler items are excluded from scoring. The higher the composite score, the higher the level of optimism. Cronbach’s alpha was 0.77 at the time of the adopting the instrument (22) and 0.71 in the current study.

#### 2.3.2. Emotional intelligence

The authors measured participants’ emotional intelligence using the Wong and Law Emotional Intelligence Scale (WLEIS) (10), which is translated and verified by Jung (23). This instrument consists of 16 items and is structured into four subscales: self-emotion appraisal, others’ emotional appraisal, use of emotions, and regulation of emotions. It is rated on a 7-point Likert scale. The score ranges from 15 to 112, with higher scores indicating higher emotional intelligence. Cronbach’s alpha was 0.87 at the time of adaptation of the instrument (23) and 0.89 in the current study.

#### 2.3.3. Self-directed learning competency

An instrument developed by Williamson to measure self-directed learning competency (24) translated and validated by Jo et al. (25), was used to measure self-directed learning competency. This instrument is rated on a 5-point Likert scale and comprises 30 items structured into five subscales: cognitive abilities (7 items), learning strategies (4 items), learning activities (5 items), assessment (5 items), and interpersonal skills (9 items). Higher scores indicate higher self-directed learning competency. Cronbach’s alpha was 0.91 to 0.95 at the time of instrument adaptation (25) and 0.90 in this study.

#### 2.3.4. Academic resilience

The Academic Resilience Scale, developed by Kim (26), was used to measure academic resilience. The instrument is structured into six subscales: regulation of learning, support of friends, self-control, positive attitude, task responsibility, and parental support; it has 29 questions. Each item is rated on a 5-point Likert scale ranging from 1 = strongly disagree to 5 = strongly agree, and higher scores indicate a higher level of academic resilience. Cronbach’s alpha ranged between 0.72 and 0.78 when the instrument was developed and 0.91 in the current study.

### 2.4. Data analysis

The collected data were analyzed using the SPSS/WIN 26.0 program. The general characteristics were derived using percentages, mean, and standard deviation, and the relationships between optimism, emotional intelligence, self-directed learning competency, and academic resilience were measured using an independent t-test, analysis of variance, Tukey test, and Pearson’s correlation coefficient. The assumptions of regression analysis were tested to verify if the data were suitable for regression analysis. The Durbin-Watson value of 1.729–2.141, tolerance of 0.501–0.915, variance inflation factor of 1.092–1.997, and the residual plot confirming the homogeneity of variance indicated that the data satisfied the assumptions of the independence of residuals, normal distribution, equal variance of dependent variables, and multicollinearity.

Baron et al.’s mediation analysis method was used to verify the model (27). In step 1, using self-directed learning competency as a mediator, it was examined if the independent variables, optimism and emotional intelligence, were statistically significant to the level of self-directed learning competency. Step 2 examined if the independent variables had a statistically significant effect on the dependent variable, academic resilience. In step 3, optimism and emotional intelligence (independent variables) and self-directed learning competency (mediator variable) were simultaneously added to the regression equation to verify if optimism, emotional intelligence, and self-directed learning competency affected academic resilience individually. The Sobel test was performed using the standard error between an unstandardized coefficient and another unstandardized coefficient to verify the significance of the mediating effect of self-directed learning competency (28).

### 2.5. Ethical considerations and data collection

This study was conducted after obtaining ethical approval (WKIRB-202106-SB-033) from the Institutional Review Board of the university with which the primary investigator is affiliated. Data were collected after obtaining consents from the participants by explaining the study’s purpose and procedure, and informing them that their anonymity and confidentiality of data will be maintained. Data were collected using Google Survey only for participants who voluntarily agreed to participate in the study. The information on research purpose, collection of personal information, and the principle of confidentiality were displayed on the first page of the Google questionnaire link to allow voluntary participation. In addition, after the survey was completed, the results of participants’ survey responses were automatically processed through the computer system so that they were not personally identifiable. Data were collected between September 30 and November 15, 2021.

## 3. Results

### 3.1. Differences in academic resilience according to participants’ general characteristics

The average age of the participants was 22.25 ± 2.05 years, with 161 females (82.6%) and 34 males (17.4%). There were 45 first-year students (23.1%), 39 s-year students (29.0%), 65 third-year students (33.3%), and 46 fourth-year students (23.6%). Regarding socioeconomic status, 143 (73.3%), 31 (15.9%), and 21 (10.8%) participants indicated it to be middle, high, and low, respectively. Most participants perceived their stress regarding school life as moderate (*n* = 106, 54.4%), followed by those who reported high (*n* = 53, 27.2%), and low (*n* = 6, 18.5%) levels of stress. Among the participants, the subjective health status was moderate for 94 (48.2%), good for 72 (36.9%), and bad for 29 (14.9%). Overall, 93 participants were satisfied with their major (47.4%), 89 were neutral (45.6%), and 13 were dissatisfied (6.7%). The most common responses to the question regarding the motivation for applying to the nursing department were in the order of interest and aptitude (*n* = 96, 49.2%), high employment rate (*n* = 47, 24.1%), others’ suggestions (*n* = 30, 15.4%), and specialized profession (*n* = 22, 11.3%).

The analysis of differences in academic resilience, according to participants’ general characteristics, showed significant variations based on the degree of school life stress (*F* = 8.83, *p* < 0.001), subjective health status (*F* = 10.67, *p* < 0.001), and satisfaction with the academic major (*F* = 15.04, *p* < 0.001). The post-hoc analysis revealed lower academic resilience among participants with high or moderate stress from school life compared to those with low stress from school life, participants with moderate or bad subjective health status compared to those with good subjective health status, and participants who were dissatisfied with their academic major compared to those who were satisfied (Table 1).

**Table 1:** Differences of academic resilience according to participants’ general characteristics (*N* = 195).

Characters	Categories	n (%) or Mean ± SD	Academic resilience		
			Mean ± SD	t or F	*p* Tukey
Age (year)			22.25 ± 2.05		
Gender	Female	161 (82.6)	4.00 ± 0.47	−1.48	0.142
	Male	34 (17.4)	3.86 ± 0.62		
Academic year	First	45 (23.1)	3.98 ± 0.55	1.53	0.208
	Second	39 (20.0)	3.86 ± 0.50		
	Third	65 (33.3)	3.96 ± 0.46		
	Fourth	46 (23.6)	4.09 ± 0.49		
Economic status	High	31 (15.9)	4.08 ± 0.55	0.89	0.414
	Middle	143 (73.3)	3.96 ± 0.48		
	Low	21 (10.8)	3.918 ± 0.54		
School life stress	High^a^	53 (27.2)	3.84 ± 0.52	8.83	<0.001
	Middle^b^	106 (54.4)	3.94 ± 0.49		a,b < c
	Low^c^	36 (18.5)	4.26 ± 0.36		
Subjective health status	Good^a^	72 (36.9)	4.16 ± 0.47	10.67	<0.001
	Moderate^b^	94 (48.2)	3.90 ± 0.45		a > b,c
	Bad^c^	29 (14.9)	3.73 ± 0.58		
Satisfaction with major	Satisfied^a^	93 (47.7)	4.14 ± 0.44	15.04	<0.001
	Neutral^b^	89 (45.6)	3.86 ± 0.46		a,b > ca > b
	Dissatisfied^c^	13 (6.7)	3.52 ± 0.67		
Motivation for applying to nursing	Interest, aptitude	96 (49.2)	4.02 ± 0.44	0.69	0.557
	High employment rate	47 (24.1)	3.93 ± 0.56		
	Others’ suggestions	30 (15.4)	3.91 ± 0.61		
	Specialized profession	22 (11.3)	3.92 ± 0.45		

“a, b, c” indicate the results of Tukey post hoc tests.They are just symbols. p and Tukey.

### 3.2. Participants’ levels of optimism, emotional intelligence, self-directed learning competency, and academic resilience

The mean optimism was 3.59 ± 0.6 out of 5, mean emotional intelligence was 5.24 ± 0.79 out of 7, mean of self-directed learning competency was 3.80 ± 0.48 out of 5, and mean academic resilience was 3.97 ± 0.50 out of 5. The scores of the subscales for emotional intelligence were the highest for self-emotion appraisal at 5.67 ± 0.93 and the lowest for regulation of emotions at 4.59 ± 1.16. The mean score of self-directed learning competency by subscale was the highest for cognitive abilities and interpersonal skills at 4.04 ± 0.54 and 4.04 ± 0.53, respectively, and the lowest for learning activities at 3.05 ± 0.81. The mean score of academic resilience by subscale was the highest for self-control at 4.22 ± 0.47 and lowest for parental support at 3.75 ± 0.85 (Table 2).

**Table 2:** Levels of optimism, “Emotional intelligence”, “Self-directed learning competency”, and “Academic resilience” (*N* = 195).

Variables	Min	Max	Mean	*SD*
Optimism	1.50	4.83	3.59	0.66
Emotional intelligence	1.13	7.00	5.24	0.79
Self-emotion appraisal	1.00	7.00	5.67	0.93
Other-emotion appraisal	1.50	7.00	5.55	1.03
Emotional utilization	1.00	7.00	5.16	1.19
Regulation of emotions	1.00	7.00	4.59	1.16
Self-directed learning competency	2.33	5.00	3.80	0.48
Cognitive abilities	2.00	5.00	4.04	0.54
Learning strategy	1.00	5.00	3.92	0.67
Learning activity	1.00	5.00	3.05	0.81
Evaluation	1.20	5.00	3.67	0.68
Interpersonal skills	2.11	5.00	4.04	0.53
Academic resilience	2.03	4.93	3.97	0.50
Learning control	1.00	5.00	3.91	0.76
Friend support	1.60	5.00	3.90	0.76
Self-control	2.50	5.00	4.22	0.47
Positive attitude	1.60	5.00	3.86	0.68
Task responsibility	2.25	5.00	4.14	0.64
Parental support	1.25	5.00	3.75	0.85

### 3.3. Correlations between optimism, emotional intelligence, self-directed learning competency, and academic resilience

Table 3 show the results of the correlation analysis of participants’ optimism, emotional intelligence, self-directed learning competency, and academic resilience. Academic resilience positively correlated with self-directed learning competency (*r* = 0.66, *p* < 0.001), optimism (*r* = 0.55, *p* < 0.001), and emotional intelligence (*r* = 0.61, *p* < 0.001). Self-directed learning competency also correlated positively with optimism (*r* = 0.31, *p* < 0.001) and emotional intelligence (*r* = 0.62, *p* < 0.001).

**Table 3:** Correlations between optimism, emotional intelligence, self-directed learning competency, and academic resilience (*N* = 195).

Variables	Self-directed learning competency	Optimism	Emotional intelligence
		*r* (*p*)	
Academic resilience	0.66 (< 0.001)	0.55 (< 0.001)	0.61 (< 0.001)
Self-directed learning competency	1	0.31 (< 0.001)	0.62 (< 0.001)
Optimism		1	0.48 (< 0.001)

### 3.4. The mediating effect of self-directed learning competency on the relationship between optimism and academic resilience

**Figure 1:**
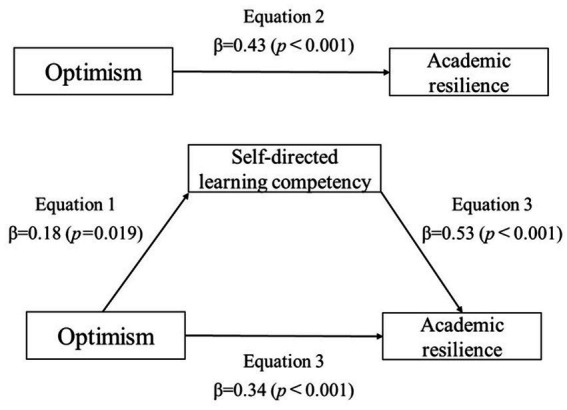
Mediating effect of self-directed learning competency on the relationship between optimism and academic resilience.

The model was analyzed to verify the mediating effect of self-directed learning competency on the relationship between optimism and academic resilience, based on Baron et al.’s procedure. School life stress, subjective health status and satisfaction with major, which showed significant differences in academic resilience, were controlled to examine the mediated effects. In step 1, optimism (independent variable) had a significant positive correlation with self-directed learning competency (mediator variable) (*β* = 0.18, *p* = 0.019). In step 2, optimism showed a significant positive correlation with academic resilience (dependent variable) (*β* = 0.43, *p* < 0.001). In step 3, optimism and self-directed learning competency were simultaneously added to the regression model. Self-directed learning competency significantly affected academic resilience (*β* = 0.53, *p* < 0.001), and optimism also affected academic resilience considerably. However, the impact was less (*β* = 0.43 to *β* = 0.34) compared to that observed in step 2. In other words, self-directed learning competency had a partial mediating effect on the relationship between optimism and academic resilience. The explanatory power of optimism for academic resilience was 33.8% (*F* = 42.16), and the explanatory power of optimism and self-directed learning competency for academic resilience increased to 56.7% (*F* = 82.28), indicating the significant effect of self-directed learning competency on academic resilience (Table 4 and Figure 1). The Sobel test was performed to verify the significance of such a mediating effect. The *Z*-value was 2.29 (*p* = 0.022), confirming a partial mediation effect of self-directed learning competency on the relationship between optimism and academic resilience.

**Table 4:** Mediating effect of self-directed learning competency on the relationship between optimism and academic resilience (*N* = 195).

Step	Direction	*B*	SE	*β*	*t*	*p*	Adj. *R*^2^	*F*	*p*
1	Optimism → Self-directed learning competency	0.13	0.05	0.18	2.37	0.019	0.181	5.60	0.019
2	Optimism → Academic resilience	0.33	0.05	0.43	6.49	<0.001	0.338	42.16	<0.001
3	Optimism → Academic resilience	0.26	0.04	0.34	6.21	<0.001	0.567	82.28	<0.001
	Self-directed learning competency → Academic resilience	0.56	0.06	0.53	10.00	<0.001			

Sobel test *Z* = 2.29, *p* = 0.022.

### 3.5. The mediating effect of self-directed learning competency on the relationship between emotional intelligence and academic resilience

**Figure 2:**
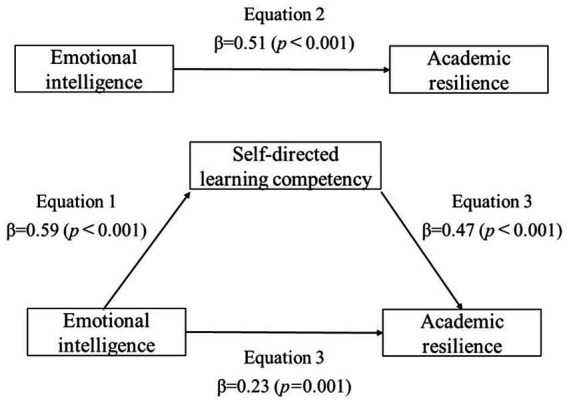
Mediating effect of self-directed learning competency on the relationship between emotional intelligence and academic resilience.

The model was analyzed to verify the mediating effect of self-directed learning competency on the relationship between emotional intelligence and academic resilience, using Baron et al.’s procedure (27). School life stress, subjective health status and satisfaction with major, which showed significant differences in academic resilience, were controlled to examine the mediated effects. In step 1, emotional intelligence (independent variable) had a significant positive correlation with self-directed learning competency (mediator variable) (*β* = 0.59, *p* < 0.001). In step 2, emotional intelligence showed a significant positive correlation with academic resilience (dependent variable) (*β* = 0.51, *p* < 0.001). In step 3, emotional intelligence and self-directed learning competency were simultaneously added to the regression model. Self-directed learning competency had a significant impact on academic resilience (*β* = 0.47, *p* < 0.001), and emotional intelligence also had a considerable effect on academic resilience. The influence was reduced (*β* = 0.51 to *β* = 0.23) compared to that seen in step 2. In other words, self-directed learning competency partially mediated the relationship between emotional intelligence and academic resilience. The explanatory power of emotional intelligence for academic resilience was 38.0% (*F* = 57.60), and the explanatory power of emotional intelligence and self-directed learning competency for academic resilience increased to 50.6% (*F* = 60.53), indicating the significant impact of self-directed learning competency on academic resilience (Table 5 and Figure 2). The Sobel test was performed to verify the significance of the mediating effect. The *Z*-value was 5.53 (*p* < 0.001), confirming a partial mediation effect of self-directed learning competency on the relationship between emotional intelligence and academic resilience.

**Table 5:** Mediating effect of self-directed learning competency on the relationship between emotional intelligence and academic resilience (*N* = 195).

Step	Direction	*B*	SE	*β*	*t*	*p*	Adj. *R*^2^	*F*	*p*
1	Emotional intelligence → Self-directed learning competency	0.35	0.04	0.59	9.00	<0.001	0.411	80.94	<0.001
2	Emotional intelligence → Academic resilience	0.32	0.04	0.51	7.59	<0.001	0.380	57.60	<0.001
3	Emotional intelligence → Academic resilience	0.15	0.05	0.23	3.27	0.001	0.506	60.53	<0.001
	Self-directed learning competency → Academic resilience	0.49	0.07	0.47	6.98	<0.001			

Sobel test *Z* = 5.53, *p* < 0.001.

## 4. Discussion

This descriptive survey study aimed to identify factors that affect the academic resilience of nursing students, focusing on optimism, emotional intelligence, and self-directed learning competency.

The study participants’ academic resilience averaged 3.97 out of 5, which is higher than the 3.79 reported by Hwang and Shin (7), 3.87 reported by Kim and Lee (6), and 3.27 reported by Lee and Jang (19), which used the same instrument. A rapid change in the academic resilience of nursing students after the COVID-19 pandemic can be inferred from the results of the current and previous studies. The results of studies conducted by Kim and Lee (6) and Hwang and Shin (7) prior to the pandemic indicate that nursing students exhibit relatively constant academic resilience. However, Lee and Jang reported a drastic reduction in academic resilience (19) in 2020, when education was imparted using non-face-to-face classes due to the pandemic. The level of academic resilience increased again in the current study, which was conducted in 2021. This can be attributed to the stable operation of in-person classes and students’ adjustment to them. It can be interpreted that academic resilience, defined as individual ability that is distinct from that of others in achieving academic success even under challenging circumstances (4, 5), improved remarkably as the pandemic caused a paradigm shift in the educational system. An international study that examined the academic resilience of graduate and undergraduate students after the COVID-19 outbreak reported that students’ academic resilience had increased, and that they demonstrated a strong will to overcome difficult situations and cope with challenges, and were able to recognize themselves as individuals who can recover from hardships (29); these findings support the current study’s results. The fact that self-control yielded the highest score (4.22) among the subscales of academic resilience in this study also indicates the situation that prevailed during the pandemic period.

The result of significantly lower academic resilience among participants with high levels of stress from school life and those who were dissatisfied with their academic major was similar to that of a preceding study (30). Academic stress and dissatisfaction have been shown to negatively impact academic satisfaction, specifically online studies (31), and thereby causing academic failure. Therefore, attempts to explore the use of academic resilience as an alternative to maximize students’ academic achievement in the context of the heavy increase in quantitative and qualitative online and non-face-to-face education are considered meaningful.

The correlation analysis between the study variables showed that the higher the optimism, emotional intelligence, and self-directed learning competency, the higher will be academic resilience; this result has been confirmed by several prior studies. Individuals with high levels of optimism are more likely to be proactive in achieving their goals and gain satisfaction while working toward them, and optimism affects academic achievement and learning engagement (32, 33). Additionally, optimism, academic resilience, and satisfaction with one’s academic major are significantly correlated with one another (6). Emotional intelligence is associated with high academic achievement (16, 34). Prior research has also confirmed the relationship between emotional intelligence, academic resilience, and self-regulation (35), as well as the correlation between positive psychological resources and academic resilience (36). Self-directed learning competency has been shown to correlate with academic self-efficacy (15, 37) and positive emotions (2), supporting the study results.

In this study, optimism, emotional intelligence, and self-directed learning competency were found to affect academic resilience. Self-directed learning competency was also found to partially mediate the relationship between optimism and academic resilience and between emotional intelligence and academic resilience. These results confirm that optimism and emotional intelligence influence academic resilience not only directly, but also indirectly through self-directed learning competency. Therefore, interventions that improve self-directed learning competency as a strategy to enhance optimism and emotional intelligence for promoting academic resilience are needed. Self-directed learning involves establishing learning objectives and plans and adopting cognitive, motivational, and behavioral strategies effectively, which is essential for successful learning by immersing oneself in learning (38, 39). Meanwhile, self-directed learning competency is an important determinant of academic achievement (40) and is associated with low academic burnout (41). It has a mediating effect on the relationships between various variables, such as emotional intelligence and creative performance (42), nursing students’ caring and resilience (43), learning motivation and learning flow (44), growth mindset and perceived academic achievement (45), and undergraduates’ level of career decisions and learning satisfaction (46). These findings demonstrate that self-directed learning competency plays an important role in enhancing academic achievement and success.

Academic achievement is not determined by specific factors but rather through the interaction of various factors, including demographic, psycho-emotional, academic, and environmental factors. Even after obtaining a nursing license, lifelong learning is central to the clinical field of nursing (47), and this involves continuous professional development, which is imperative to update nurses’ knowledge and skills (48). Therefore, nursing students must establish effective learning strategies to complete theoretical and practical training courses and fulfill their role as nurse practitioners after graduation; this is also a pragmatic way of adjusting with the paradigm shift in education following the COVID-19 pandemic. The self-directed learning competency identified in this study provides the direction for future nursing education, and it is necessary to reflect it in the educational content and nursing methods and use it actively. Therefore, in order to enhance self-directed learning competency, an educational environment in which repetitive learning is possible is established, and interactions between instructors and learners and between learners are facilitated even in an online environment. It is necessary to develop and apply a curriculum that learners set goals for themselves, establish plans to achieve them, and practice them. Moreover, based on the research result that learners’ self-directed learning competency can be well established in e-learning when learners recognize a sense of presence in the learning process (49), a teaching-learning method for online classes focusing on presence should be developed. Considering the characteristics of nursing education in which harmony between theory and practice is important, active use of virtual simulation and extended simulation can be considered.

This study is significant in that it confirms the role of self-directed learning competency in enhancing academic resilience. However, this study has several limitations. The cross-sectional design and convenience sampling limits generalization of the results and the causal relationship between variables could not be determined. Through previous studies, the authors were able to confirm significant correlations among variables such as optimism, emotional intelligence, self-directed learning competency, and academic resilience; and a significant mediating effect of self-directed learning competency (2, 6, 7, 16–18). Therefore, we tried to explore how the relationship between these variables changed under the non-face-to-face online class environment following the COVID-19 pandemic. We reached similar results to previous studies. In particular, based on the previous results that online classes enhance learners’ self-directed learning ability which influences nursing students’ academic achievement (13, 14), we aimed to examine the role of self-directed learning competency in these relationships while online classes lasted more than 2 years. We reached similar results to previous studies. However, there is a limitation in that the structural equation that confirms the relationship between each variable has not been analyzed. Hence, it needs to perform structural equation modeling to compare two mediation models, one with optimism and the other with emotional intelligence and to control for other effects. Additionally, it is necessary to conduct longitudinal studies and studies on intervention programs for improving self-directed learning competency. Repeated studies are required to confirm the impact of various factors on academic resilience.

## 5. Conclusion

This descriptive survey study identified factors that affect nursing students’ academic resilience, centered on optimism, emotional intelligence, and self-directed learning competency. The study results showed that when optimism and emotional intelligence are higher, self-directed learning competency is higher, and so is academic resilience. In addition, optimism, emotional intelligence, and self-directed learning competency affect academic resilience, and self-directed learning competency mediates the relationship between optimism, emotional intelligence, and academic resilience. Changes in the education system are inevitable due to the clinical setting and the impact of the COVID-19 pandemic. This study suggests strengthening nursing students’ positive attributes and self-directed learning competency as a strategy to prepare for changes in education; improving the nursing curriculum and teaching methods to achieve this seems imperative in the future.

## Data availability statement

The datasets presented in this article are not readily available because the data are not publicly available due to participants’ privacy under the IRB approval. Requests to access the datasets should be directed to KK, konhee@ewha.ac.kr.

## Ethics statement

The studies involving human participants were reviewed and approved by the Institutional Review Board of Wonkwang University. The participants provided their written informed consent to participate in this study.

## Author contributions

EH and KK conceptualized, designed this study, collected the data, provided resources, and drafted the manuscript. EH analyzed the data and prepared figures and tables. All authors read, approved the final manuscript, and agreed to the published version of manuscript.

## Funding

This work was supported by the Wonkwang University in 2022.

## Conflict of interest

The authors declare that the research was conducted in the absence of any commercial or financial relationships that could be construed as a potential conflict of interest.

## Publisher’s note

All claims expressed in this article are solely those of the authors and do not necessarily represent those of their affiliated organizations, or those of the publisher, the editors and the reviewers. Any product that may be evaluated in this article, or claim that may be made by its manufacturer, is not guaranteed or endorsed by the publisher.
